# Effects of Nesting Material Provision and High-Dose Vitamin C Supplementation during the Peripartum Period on Prepartum Nest-Building Behavior, Farrowing Process, Oxidative Stress Status, Cortisol Levels, and Preovulatory Follicle Development in Hyperprolific Sows

**DOI:** 10.3390/antiox13020210

**Published:** 2024-02-07

**Authors:** Hyeonwook Shin, Juho Lee, Junsik Kim, Geonil Lee, Jinhyeon Yun

**Affiliations:** Department of Animal Science, College of Agriculture and Life Sciences, Chonnam National University, Gwangju 61186, Republic of Korea; sinhu97@gmail.com (H.S.); juho6541@gmail.com (J.L.); kk94032683@gmail.com (J.K.); clerk123@naver.com (G.L.)

**Keywords:** antioxidants, maternal characteristics, nesting material, pro-oxidants, reactive oxygen species

## Abstract

Hyperprolific sows often experience increased oxidative stress during late gestation and lactation periods, which can adversely affect the farrowing process and overall lactation performance. This study examines the influence of providing a coconut coir mat (CCM; 1 × 1 m) as nesting material, supplementing high-dose vit-C (HVC; 20% vit-C, 10 g/kg feed) as an antioxidant, or both on maternal behavior, the farrowing process, oxidative status, cortisol levels, and preovulatory follicle developments in sows with large litters. In total, 35 sows (Landrace × Yorkshire; litter size 15.43 ± 0.27) were allocated to the following four treatment groups: control (*n* = 9, basal diet), vit-C (*n* = 8, basal diet + HVC), mat (*n* = 10, basal diet + CCM), and mat + vit-C (*n* = 8, basal diet + HVC + CCM). A post-hoc analysis showed that compared with sows that were not provided CCM, mat and mat + vit-C groups demonstrated increased durations of nest-building behavior during the period from 24 h to 12 h before parturition (*p* < 0.05 for both), reduced farrowing durations, and decreased intervals from birth to first udder contact (*p* < 0.01 for both). The mat group exhibited lower advanced oxidation protein product (AOPP) levels during late gestation and lactation periods than the control group (*p* < 0.05). Sows with HVC supplementation showed longer farrowing durations than those without HVC supplementation (*p* < 0.0001). The vit-C group had higher salivary cortisol levels on day 1 after farrowing than the other treatment groups (*p* < 0.05). Furthermore, the follicle diameters on day 3 after weaning in the vit-C group tended to be smaller than those in the control group (*p* = 0.077). HVC supplementation prolonged farrowing and increased the physiological stress on postpartum, and no advantageous effects on maternal behavior and developmental progression of preovulatory follicles were observed. Hence, alternative solutions beyond nutritional approaches are required to address increased oxidative stress in hyperprolific sows and secure their welfare and reproductive performance. The present results substantiated the positive impact of providing CCM as nesting material for sows with large litters on nest-building behavior and the farrowing process, which could mitigate the deleterious consequences induced by peripartum physiological and oxidative stress.

## 1. Introduction

Over the last three decades, the pig industry has experienced a drastic rise in the size of sow litters [1]. However, this increase in litter size has been accompanied by prolonged farrowing, resulting in increased stillbirth rates and diminished piglet vitality [2]. The enlarged litter size might be associated with physiological and oxidative stress due to the increased energy requirements for fetal growth and milk production during gestation and lactation [3]. This, in turn, can disrupt maternal hormone secretion and elevate the risk of reproductive disorders, such as mummification, preterm labor, miscarriage, and inhibition of preovulatory follicular development [3,4].

There is a growing interest in addressing the oxidative stress of mammals via supplementation of antioxidants, such as plant extracts, vitamin E, vitamin C, and selenium [4,5,6,7,8]. Despite variations in the impact of antioxidants depending on differences in animal species and physiological status [9], vitamin C consistently acts as a one- or two-electron donor for free radicals, thereby minimizing the variability of its effects [10,11]. Uchio et al. [12] demonstrated that the administration of high-dose vitamin C (HVC, 200 mg/kg daily for two months) reduced oxidative stress by elevating intracellular levels of other antioxidants, including superoxide dismutase and glutathione, in the splenocytes of dexamethasone-treated mice. Hyperprolific sows experience elevated oxidative stress with increasing litter sizes; hence, the supplementation of HVC in sows with large litters should be considered to counteract it [8].

An alternative strategy for modulating oxidative stress in sows involves providing environmental enrichment, such as adjusting the thermal environment and ensuring the availability of nesting materials and sufficient space [13,14]. In the modern pig industry, peripartum sows often experience stress due to confinement in farrowing crates and absence of nesting materials [15] that impedes their maternal instinct for nest-building behavior (NB). Captive sows with large litters may experience increased oxidative and physiological stress [4,13,14], which hinders the maternal behavior of peripartum sows due to the accumulation of reactive oxygen species (ROS) [16]. Hence, providing appropriate nesting materials may be beneficial in addressing the issue of inhibited instinctive behavior in sows due to increased oxidative stress, which consequently affects their farrowing and reproductive performance [17,18].

A previous study conducted by our research group demonstrated that sows with large litters (over 14 piglets) exhibit higher oxidative stress than those with small litters, which is associated with reduced NB and reproductive performance [4]. Therefore, our current study aims to investigate the potential mitigation of oxidative stress in sows with large litters by supplementing HVC as an antioxidant. Additionally, the impact of providing CCM as an inducer for prepartum NB is examined by evaluating its effect on the management of oxidative stress and enhancement of reproductive performance in sows with large litters.

## 2. Material and Methods

This study was conducted at a commercial farm with an 8000-head pig unit in southern South Korea between June and August 2022. All the experimental animal procedures were reviewed and approved by the Chonnam National University Institutional Animal Care and Use Committee (reference number: CNU IACUC-YB-2021-167).

### 2.1. Animals and Management

A total of 35 sows (Landrace × Yorkshire; parity, 1.91 ± 0.28) from three batches at expected farrowing intervals of 7 days were used in this study. The litter size of the experimental sows was more than 13 because the number of their functional teats was between 13 and 14. During pregnancy, the sows were housed in groups of 25 to 29 per pen (5.75 × 10.5 m), and 1 kg of a standard gestational diet (3.3 Mcal of digestible energy per kg, including 12.8% crude protein, 3.7% crude fat, 4.1% crude fiber, 4.6% crude ash, 0.8% calcium, and 0.4% phosphorus) was provided twice a day at 08:30 and 16:00 with ad libitum access to water. Approximately 4–5 days before the expected farrowing date, the sows were moved to farrowing pens (2.3 × 2.4 m) with fully concrete slatted floors. The sows were maintained in loose housing conditions during the entire parturition and lactation period. Housing conditions and pen features were as in our previous study [4]. The standard lactation diet (1 kg; 3.5 Mcal of digestible energy per kg, including 14.7% crude protein, 3.2% crude fat, 3.0% crude fiber, 5.2% crude ash, 0.9% calcium, and 0.5% phosphorus) was provided automatically twice daily at 09:30 and 15:00 until the farrowing date. After farrowing, the feed was gradually increased by 1 kg per day until the total feed amount reached 7 kg. Within 48 h postpartum, piglets were cross-fostered to standardize the number of piglets on each experimental sow. After approximately 4 weeks of lactation, the sows were transported to a breeding house and housed in individual stalls (0.65 × 2.1 m).

### 2.2. Experimental Design

**Figure 1:**
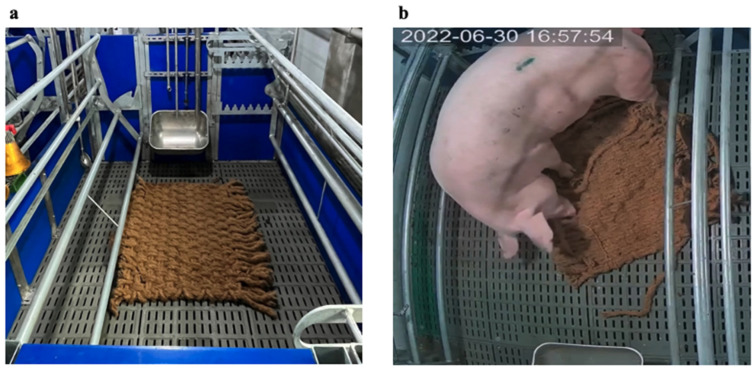
(**a**) Adjustable farrowing crate in open condition (pen size = 2.3 × 2.4 m) with a coconut coir mat (1.0 × 1.0 m). (**b**) A sow performing nest-building behavior using a coconut coir mat.

All the sows were randomly assigned to one of the following four experimental groups: 1. control (*n* = 9, basal diet); 2. vit-C (*n* = 8, basal diet + HVC [20% vit-C, 10 g/kg feed]); 3. mat (*n* = 10, basal diet + coconut coir mat [CCM; 1 × 1 m] provided on the slatted floor); and 4. mat + vit-C (*n* = 8, basal diet + HVC [20% vit-C, 10 g/kg feed] + CCM ([1 × 1 m]) provided on the slatted floor). The CCM (ECOLJS Co., Cheongju, Republic of Korea) was placed at the center of the farrowing pen without affixing (Figure 1) so that the sows could freely manipulate it 4 days before the expected date of parturition. After parturition, 2/3 of the CCM was removed to prevent loosening of the mat and debris from falling through the slatted floor. The rest of the CCM was placed under a heat lamp to guide the piglets to a safe area. The diet was supplemented with HVC (DaOne Chemical Co., Siheung, Republic of Korea) when the feed was provided using the automatic feeder at 09:30 and 15:00, starting from 4 days before the expected farrowing date until 2 weeks after parturition.

### 2.3. Data Collection

#### 2.3.1. Saliva Collection and Assays

##### Saliva Collection

Saliva samples were collected using a synthetic swab (Salivette^®^ Cortisol; Sarstedt, Germany) at the following times: 4 days before the farrowing date and 1, 7, and 28 days after farrowing. Saliva samples were collected 1 h after the morning feeding. The swab was fixed with forceps and placed around the sow’s molars for 1 min to induce chewing. All the saliva samples were stored at −50 °C in a deep freezer on the farm for 1 month, then transported to the laboratory in a portable freezer at −20 °C, and stored at −80 °C in a deep freezer until analysis.

##### Assay for Oxidative Stress Parameters

For the analysis of levels of advanced oxidation protein products (AOPP), the saliva samples were centrifuged at 1000× *g* for 10 min, and the supernatants were analyzed using an enzyme-linked immunosorbent assay (ELISA) kit (AOPP ELISA kit; MBS028634, San Diego, CA, USA) according to the manufacturer’s instructions. For the analysis of hydrogen peroxide (H_2_O_2_), Trolox equivalent antioxidant capacity (TEAC), and tumor necrosis factor-alpha (TNF-α) levels, the saliva samples were centrifuged at 10,000× *g* for 10 min, and the supernatants were analyzed using an ELISA kit (OxiSelect™ Hydrogen Peroxide Assay [MBS169261, San Diego, CA, USA], OxiSelect™ TEAC Assay Kit [MBS169313, San Diego, CA, USA], and Porcine TNF-α DuoSet ELISA [DY690B, Minneapolis, MN, USA], respectively) according to the manufacturer’s instructions. The sensitivity values of the AOPP, H_2_O_2_, and TEAC assays were 1.0 μmol/L, 0.8 μM g/mol, and 250.29 g/mol, respectively. TNF-α had a detection limit of 31.3 pg/mL.

##### Cortisol Assay

For the analysis of cortisol, the saliva samples were centrifuged at 1000× *g* for 10 min, and the supernatants were analyzed using an ELISA kit (Salimetrics^®^ Cortisol Enzyme Immunoassay Kit; 1-3002, Carlsbad, CA, USA) according to the manufacturer’s instructions. The sensitivity of the cortisol assay was 0.018 μg/dL.

#### 2.3.2. Colostrum Collection and Assays

More than 25 mL of the colostrum samples were collected within 3 h of parturition in a 50 mL conical tube (SPL, Pocheon, Republic of Korea). The samples were stored at −50 °C in a deep freezer on the farm for 1 month, then transported in a portable freezer at −20 °C, and stored at –80 °C in a deep freezer until analysis. For the analysis of oxytocin and prolactin concentrations, the colostrum samples were centrifuged at 1000× *g* for 20 min, and the skimmed milks were analyzed using an ELISA kit (OT ELISA Kit [E-EL-0029, Houston, TX, USA] and Pig Prolactin ELISA Kit [MBS2087044, San Diego, CA, USA], respectively) according to the manufacturer’s instructions. The sensitivity values of the oxytocin and prolactin assays were 9.38 pg/mL and 6.1 pg/mL, respectively. For the analysis of immunoglobulin (Ig) A, IgG, and IgM concentrations, the colostrum samples were centrifuged at 1000× *g* for 20 min, and the skimmed milks were analyzed using an ELISA Kit (Pig IgA ELISA Kit [E101-102, Montgomery, TX, USA], Pig IgG ELISA Kit [E101-104, Montgomery, TX, USA], and Pig IgM ELISA Kit [E101-117, Montgomery, TX, USA], respectively) with a detection range of 1.37–1000 ng/mL.

#### 2.3.3. Behavioral Observation

Video recordings of the sows were taken for 48 h (24 h each, before and after parturition) to assess prepartum NB and the farrowing process of sows using internet protocol (IP) cameras (HN0-E60; Hanwha Techwin, Changwon, Republic of Korea) with 1920 × 1080-pixel resolution at 30 FPS. One camera per two pens was installed on a wooden bar 1.5 m above the sows’ heads. A trained researcher used a media player (PotPlayer, Kakao Corp., Jeju, Republic of Korea) to observe the NB and farrowing processes of all the sows.

The start of NB was defined as active pawing, rooting, and arranging for longer than 5 s. The end of NB was defined as no pawing, rooting, and arranging for longer than 30 s. The progression of NB was tracked at 6 h intervals as the sows approached parturition. Pawing was defined as scratching the floor with the front legs, rooting as nudging or pushing the floor or crate with the snout, and arranging as shifting or sorting nesting materials using the snout or mouth.

The duration of farrowing was considered as the time period between the births of the first and last piglet, including stillbirths. The birth interval, which is the time between the birth of consecutive piglets, including stillbirths, was also evaluated. Additionally, the birth-to-udder touch interval in piglets was calculated from when a piglet was born until its first contact with the sow’s udder.

#### 2.3.4. Measurement of Follicle Size

The ovarian status of the sows from the third batch was examined using transrectal ultrasound examinations (Easi-Scan: Go, 4.5 MHz–8.5 MHz, curved probe; IMV Imaging, Bellshill, Scotland, UK). The examinations were conducted daily at 13:00, starting from 3 days before ovulation until ovulation. Both ovaries were scanned separately during examination, and the clips were exported in MPEG-4 format for analysis. The diameters (mm) of the five largest follicles in both ovaries were measured, and the average was calculated to define follicle size. At 09:00 and 16:00 every day from 3 days after weaning, the boars were released into the aisle in front of the sow’s feeder to induce contact with the sow, and standing estrus was examined. All the sows were artificially inseminated after estrus was first detected, and if they continued to exhibit standing reflex, insemination was repeated when estrus was subsequently detected.

### 2.4. Statistical Analysis

All the data were statistically analyzed using SAS, version 9.4 (SAS Institute Inc., Cary, NC, USA, 2012). Each sow was considered as one experimental unit. Normality was tested via the UNIVARIATE procedure using the Shapiro–Wilk test. The fixed effect was the treatment, and the random effect was the farrowing batch, which had 1week intervals. Data were presented as means and standard errors of the mean (SEM).

A PROC GLIMMIX model with Lognormal distribution was fitted to assess salivary cortisol, oxidative stress parameters (H_2_O_2_, TEAC, AOPP, and TNF-α), and colostral parameters (oxytocin, prolactin, IgG, IgM, and IgA). A repeated measure test was used for the data analysis on oxidative stress parameters during prepartum and lactation periods (D−4, D1, D7, and D28). The PROC MIXED model was used to determine NB, litter weight, and follicle size. The PROC GLIMMIX model with Poisson distribution was conducted to analyze the farrowing process, litter characteristics, and periods of weaning-to-estrus and estrus-to-ovulation. Spearman’s rank correlation coefficient (r) was used to analyze correlation data.

## 3. Results

### 3.1. Oxidative Stress Parameters

The salivary oxidative stress parameters of sows from 4 days before farrowing to 28 days after farrowing are presented in Table 1. On day 4 before farrowing, the AOPP levels of the control group were higher than those of the vit-C or mat group (*p* < 0.05, Table 1). However, there was no difference when compared to the mat + vit-C group (Table 1). On day 7 after farrowing, the AOPP levels tended to be lower in sows provided with CCM than those in sows without CCM (*p* = 0.086, Table 1). Additionally, the repeated measures analysis revealed that the control group exhibited higher levels of AOPP than the mat group from 4 days before farrowing to 28 days after farrowing (*p* < 0.05, Table 1).

A post-hoc analysis showed that HVC supplementation increased the sows’ TEAC levels on day 1 after farrowing (*p* < 0.05, Table 1). However, there were no significant differences in the TEAC levels among treatment groups in the other sampling periods and the period between −4 and 28 days.

CCM provision tended to lower the H_2_O_2_ levels on day 28 after farrowing (*p* = 0.074, Table 1) but did not alter them on the other days. Additionally, no significant differences were found in the TNF-α levels among the treatment groups during all the sampling periods.

On day 4 before farrowing, the H_2_O_2_ levels were positively correlated with the TEAC levels (r = 0.42, *p* < 0.05). During the lactation period (D1, D7, and D28), they were also correlated with the TEAC (r = 0.24, *p* < 0.05) and AOPP levels (r = 0.22, *p* < 0.05).

### 3.2. Salivary Cortisol Levels

**Figure 2:**
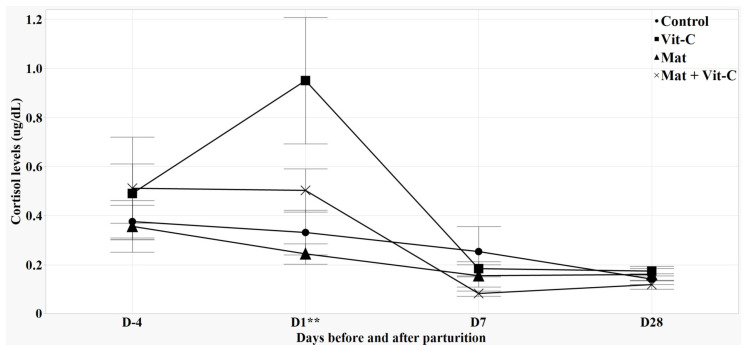
Effects of CCM provision and HVC supplementation on salivary cortisol levels of sows. All saliva samples were collected at 10:30 on each sampling day. Values represent means and standard error. ** *p* < 0.05.

The vit-C group showed higher levels of salivary cortisol than other treatment groups on day 1 after farrowing, (*p* < 0.05, Figure 2). Conversely, the post-hoc analysis showed that the sows provided with CCM tended to have lower levels of salivary cortisol than those without CCM on day 7 after farrowing (*p* = 0.060). During the lactation period (D1, D7, and D28), the salivary cortisol levels showed a correlation with the TNF-α and TEAC levels (r = 0.39, r = 0.38, respectively; *p* < 0.001) and tended to have a positive correlation with the H_2_O_2_ and AOPP levels (r = 0.17, r = 0.20, respectively; *p* < 0.10). Additionally, the salivary cortisol levels on D1 were positively correlated with farrowing duration and birth intervals (r = 0.35, r = 0.34, respectively; *p* < 0.05).

### 3.3. Colostrum Oxytocin, Prolactin, and Ig Levels

The levels of oxytocin and prolactin in the colostrum were numerically the highest in the mat group, but no significant differences were observed among the treatment groups. The sows with HVC supplementation tended to have lower IgM levels than those without HVC supplementation (*p* = 0.077, Table 2).

### 3.4. Prepartum NB

**Figure 3:**
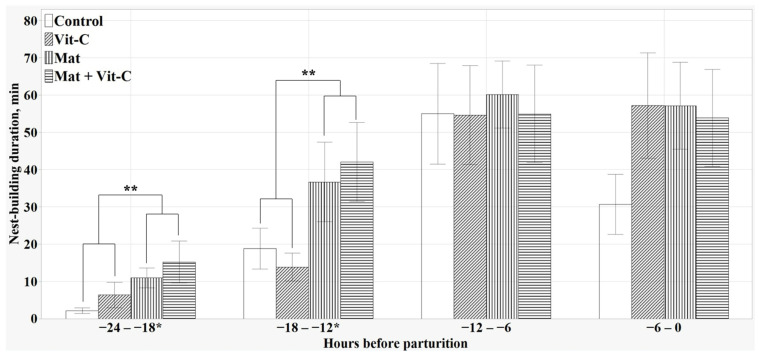
Duration of nest-building behavior every 6 h from 24 h before parturition. Values represent means and standard errors. * *p* < 0.10, ** *p* < 0.05.

The provision of CCM increased the durations of NB during the period from 24 h to 12 h before parturition when compared with those of sows without CCM (*p* < 0.05 for both, Figure 3). During the 24 h period before parturition, the sows provided with CCM showed significantly longer durations of NB than sows without CCM (means [min] ± SEM: control = 1.93 ± 0.31, vit-C = 1.95 ± 0.22, mat = 2.94 ± 0.23, mat + vit-C = 2.83 ± 0.23; *p* = 0.001). However, the frequency of NB during the 24 h period before parturition did not differ significantly among the treatment groups (means ± SEM: control = 50.56 ± 9.57, vit-C = 64.50 ± 8.48, mat = 56.70 ± 4.5, mat + vit-C = 61.50 ± 10.05; *p* = 0.585). Additionally, HVC supplementation had no significant impact on either the duration or frequency of NB during the 24 h period before parturition (*p* = 0.820, *p* = 0.220, respectively).

### 3.5. Farrowing Process and Litter Characteristics

The post-hoc analysis showed that the provision of CCM reduced the duration of farrowing and interval from birth to first udder touch by the piglet (*p* < 0.01 for both, Table 3). Conversely, the sows with HVC supplementation exhibited increased farrowing duration (*p* < 0.01, Table 3) and tended to have longer birth intervals than those without HVC supplementation (*p* = 0.078, Table 3). The total- and live-born piglet numbers did not differ among the treatment groups. However, the stillbirth frequency was significantly higher in sows provided with CCM than in those without CCM (*p* < 0.01, Table 3).

### 3.6. Follicular Development

**Figure 4:**
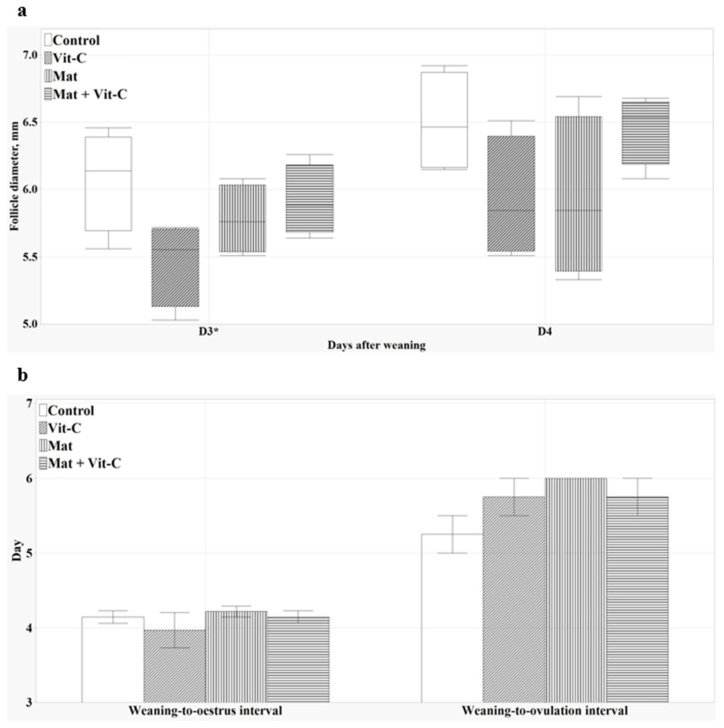
Effects of CCM provision and HVC supplementation on preovulatory follicular development (**a**) and weaning-to-oestrus and weaning-to-ovulation intervals (**b**) in sows. The diameters (in mm) of the five largest follicles on the left and right ovary were measured, and the average was calculated after weaning until ovulation. Values represent means and standard error. * *p* < 0.10.

The mean diameter of the five largest follicles on both ovaries tended to be smaller in the vit-C group than that of those in the control group 3 days after weaning (*p* = 0.077, Figure 4a). Conversely, the weaning-to-estrus and weaning-to-ovulation intervals did not differ among the treatment groups (Figure 4b).

## 4. Discussion

Hyperprolific sows are known to experience severe oxidative stress, primarily attributable to the elevated energy required for fetal development and milk production during the late gestation and lactation periods. This stress negatively affects the maternal behaviors and reproductive performances of peripartum sows [4,5]. The present study showed that the provision of CCM as nesting material in a loose-housed pen effectively promotes NB. The encouragement of instinctive behavior in prepartum pigs may alleviate the deleterious effects induced by elevated oxidative and physiological stress resulting from large litters. However, CCM provision had no discernible influence on the development of preovulatory follicles in sows after weaning.

The impact of CCM provision on counteracting the adverse effects associated with increased oxidative stress in hyperprolific sows was evaluated by comparing it to that observed by supplementation with antioxidants. However, the commercial feed used in this study already fulfilled the requirements for antioxidants, such as selenium (0.15 mg/kg) and vitamin E (44 IU/kg), according to the Korean feeding standards for swine [19]. Therefore, supplementation with HVC, a water-soluble vitamin known for its potent antioxidant properties, was implemented to mitigate the overloaded oxidative stress in hyperprolific sows during the late gestation and early lactation periods [10,11]. However, this led to prolonged farrowing and heightened physiological stress and had no advantageous effects on maternal behavior and the developmental progression of preovulatory follicles. We speculate that the pro-oxidant effect of HVC was exacerbated, as the feed contained the maximum restriction levels of selenium and vitamin E according to the Korean feeding standards for swine [19]. However, consideration of vitamin C supplementation in the feed may still be an option, since it is impossible to add additional selenium and vitamin E to the modern sow feed according to the Korean specifications [19]. Therefore, we believe that our current findings can serve as basis information for adjusting the amount of vitamin C supplementation.

The assessment of AOPP and H_2_O_2_ levels indicated that CCM provision tended to lower oxidative stress among sows with large litters during late lactation. These findings conform to those of previous studies, which demonstrated that sows may experience heightened oxidative stress, particularly during late gestation and lactation periods, especially when exposed to environmental stressors, such as the absence of nesting material and suboptimal housing conditions [13,14]. Furthermore, a positive correlation between oxidative stress status, as indicated by the increased AOPP and H_2_O_2_ levels, and cortisol levels observed in this study aligns with the observation by Rubio et al. [20] who demonstrated a positive correlation between increased stress and cortisol levels, induced by shearing in sheep, and heightened AOPP and H_2_O_2_ levels. Jarvis et al. [21] reported that prepartum sows deprived of nesting material experience elevated physiological stress due to disrupted NB than those provided with straw. Consistent with these findings, a recent study by Plush et al. [22] highlighted that supplying approximately 2 kg of straw daily until parturition extends the NB duration, which reduces plasma cortisol concentrations in sows. Consequently, the prepartum period in sows is a critical time, wherein the absence of adequate space and materials to perform NB can induce increased cortisol secretion because of the activation of the hypothalamic-pituitary-adrenal axis [23,24,25,26]. These results suggest that the provision of CCM as a nesting material may have the potential to diminish physiological stress by stimulating prepartum NB, thereby alleviating oxidative stress in sows during late gestation and lactation periods.

The increased TEAC levels on day 1 after farrowing associated with HVC supplementation, as shown in the current study, aligns with the findings of Zulueta et al. [27] who reported a strong positive correlation between the TEAC levels and vitamin C content in juice-milk beverages. This suggests the potential impact of HVC supplementation on the enhancement of overall antioxidant capacity. Furthermore, the rise in TEAC levels in response to HVC supplementation in sows implies an improved ability to counteract oxidative stress [28]. However, we did not observe significant effects of HVC supplementation on the AOPP levels throughout the experimental periods, despite the control group showing higher AOPP levels when compared separately to the vit-C or mat group on day 4 before parturition. These contrasting outcomes may be attributed to the large litter size, which induces excessive physiological and oxidative stress due to increased energy demands for fetal growth and milk production in late gestation and lactation [3]. In particular, milk production in sows continually rises after farrowing, peaking between 7 to 12 days postpartum [29,30,31]. This surge in milk production can trigger catabolic activity in the animal, leading to elevated oxidative damage at the cellular level [3,4,31]. Therefore, we speculate that the escalating oxidative stress caused by substantial milk production likely exceeds the neutralizing capacity of intrinsic antioxidants present in the feed.

TNF-α, which is produced by activated macrophages and monocytes, is an inflammatory cytokine that regulates cellular proliferation, differentiation, death, and immune responses [32,33]. A recent study explored the potential impact of providing approximately 2 cm of straw bedding once a week during late gestation and lactation periods on the TNF-α levels in sows [34]. However, such bedding materials did not influence the TNF-α levels. In contrast, Chen et al. [35] demonstrated that vitamin C supplementation could decrease the TNF-α levels in lipopolysaccharide-stimulated cells. This reduction was achieved by curbing the production of ROS, which are known to trigger the release of inflammatory cytokines. However, the present results revealed that neither providing nesting material nor supplementing vitamin C has a discernible impact on the TNF-α levels in sows. This lack of influence could potentially be attributed to the hyper-inflammatory responses observed in hyperprolific sows. These responses stem from various factors, including limited uterine capacity, prolonged farrowing, and increased synthesis of colostrum and milk during lactation [4,36] The cumulative effect of these factors might lead to the substantial release of inflammatory cytokines, possibly surpassing any anti-inflammatory potential that HVC supplementation might have held [37,38].

High cortisol levels in sows can have adverse consequences because they inhibit the release of prolactin and oxytocin [39,40,41]. This physiological disruption may lead to decreased success in the suckling of piglets and reduced average litter weight [42]. In this study, sows provided with CCM displayed a tendency toward decreased cortisol levels during early lactation. This trend might possibly be attributed to the fulfillment of their natural desire to perform prepartum NB. In contrast, HVC supplementation increased the cortisol levels on day 1 of lactation. This rise in the cortisol levels might have been due to discomfort and pain from prolonged farrowing in sows, as evidenced by the positive correlation between the cortisol levels on D1 and farrowing duration in this study. Mainau and Manteca [36] and Oliviero et al. [43] reported that prolonged farrowing causes acute stress, primarily due to pain and exhaustion, leading to increased plasma cortisol concentrations.

Additionally, the increased cortisol levels may be associated with the intake of high doses of vitamin C, which produces potential adverse effects, such as nausea, heartburn, and stomach cramps [42]. According to Heimer et al. [42], vitamin C intake exceeding 2000 mg/day increases the risk of adverse gastrointestinal effects in adult humans. Chambial et al. [44] also reported that high doses of vitamin C (2–6 g daily) can cause gastrointestinal disturbances in humans. In the current study, high-concentration vitamin C (20%) was supplemented at a rate of 20 g/day before parturition, which was then gradually increased by 10 g/day after parturition until it reached 70 g. This supplementation strategy was employed to mitigate a potential rise in oxidative stress in the hyperprolific sows during periparturient and lactation periods. However, considering sows can synthesize ascorbic acid, commonly known as vitamin C, supplementation of this vitamin may be unnecessary [45]. Hence, the vitamin C dose administered in this study might have been excessive for the sows. These findings indicate the potential risks associated with prolonged farrowing and potential gastrointestinal disturbances resulting from excessive HVC supplementation, leading to increased stress levels in sows, which emphasize the importance of balanced and carefully monitored nutritional interventions.

According to Yun et al. [17] and Wischner et al. [46], the provision of abundant nesting materials may contribute to increased oxytocin and prolactin concentrations in peripartum sows. Additionally, Yun et al. [18] reported that supplying nesting materials to sows can elevate piglet serum IgM concentrations by enhancing overall colostrum yield. Hence, we anticipated that providing CCM to prepartum sows as nesting material might increase maternal hormones and Ig yield. However, while the mat group exhibited numerically higher levels of colostrum oxytocin, prolactin, IgG, and IgM than the control group, the difference was not statistically significant. This may be attributed to temporal variation among the colostrum samples, which were collected within 3 h after parturition. In fact, the concentration of Ig in colostrum decreases drastically after the onset of parturition [47,48].

The current findings clarify the facilitation of prepartum NB in sows with large litters. The provision of CCM as nesting material to the sows housed in pens with slatted floors was notably efficacious in promoting prepartum NB when compared with just the provision of additional space, vitamin C supplementation, or both. These results signify the suitability of CCM as a nesting material owing to its edible, chewable, investigable, and destructible characteristics. Additionally, it does not fall easily into the slurry pit, thereby preventing issues with manure treatment. The active stimulation of NB in sows by the provision of CCM is supported by previous studies [49,50]. The initiation of prepartum NB is intricately linked to a discernible rise in prolactin concentration in sows [51]. Wischener et al. [46] suggested that the provision of nesting material can also potentially elevate prolactin concentrations in prepartum sows, thus contributing to the initiation of prepartum NB. The continuous activation of prepartum NB in sows relies on a delicate balance between internal feedback mechanisms and external stimuli [46]. In the present study, providing CCM as nesting material played a crucial role in offering external stimuli, addressing both the behavioral and physiological requirements for active NB in sows. This concept has been validated in previous studies [17,52,53]. Meanwhile, we found that the average number of stillbirths per litter was high in the CCM group. It is suggested that an elevated stillbirth rate has been linked to prolonged farrowing [2]. However, our current findings did not establish a clear association between the farrowing process and stillbirths. This aligns with a prior study by Yun et al. [54]. Despite these observations, we were unable to identify the reasons for the lack of beneficial effects of nesting materials on stillbirths.

The present study showed that HVC supplementation poses challenges during the farrowing phase in sows, primarily by increasing farrowing duration. This outcome can be rationalized by the notion that the excessive supplementation of vitamin C may interfere with the farrowing process by inhibiting the synthesis of prostaglandins (PGs), which are intimately related to the initiation of parturition and cervical dilation. According to Fiebich et al. [55], vitamin C has a dose-dependent inhibitory effect on the synthesis of PG, hindering the activity of cyclooxygenases in primary rat microglia. Similarly, Rosenkrans et al. [56] reported that vitamin C decreases the release of PGE and PGF2α in the endometrium of sows. Furthermore, excessive vitamin C supplementation induces gastrointestinal discomfort, which in turn, escalates cortisol levels before farrowing [42,44]. Increased cortisol may potentially inhibit the secretion of crucial maternal hormones, such as oxytocin and prolactin, thereby leading to prolonged farrowing [57,58]. Based on these findings, we speculate that the HVC supplementation in sows might have inhibited the secretion of essential hormones, resulting in prolonged farrowing duration. Nonetheless, the current findings, which reveal the absence of significant differences in the colostrum oxytocin and prolactin levels among the treatment groups, fail to substantiate the validity of this assumption. As previously explained, this is believed to stem from a potential error in the colostrum sampling method and therefore requires further research.

Excessive oxidative stress can exert detrimental effects on the integrity of oocytes and consequently, the overall reproductive processes. The present study showed that HVC supplementation tended to reduce follicle diameters compared to the control group 3 days after weaning. This might have been due to the Fenton reaction—i.e., high-dose vitamin C can act as a pro-oxidant instead of an antioxidant [59]. This reaction can potentially increase ROS production, which in turn, can trigger the apoptosis of oocytes and disrupt the delicate balance of crucial hormones such as the follicle-stimulating hormone and luteinizing hormone, both of which play critical roles in follicle growth and ovulation [60]. However, a study by Tarin et al. [61] showed the potential adverse effects of the oral administration of vitamin C (10 g/kg diet) and vitamin E (0.6 g/kg diet) in mice. High doses of these vitamins led to hypervitaminosis, which disrupts ovarian steroidogenesis and fertility. Furthermore, Kere et al. [62] demonstrated that HVC supplementation (100 µg/mL) induces apoptosis during the in vitro maturation of porcine oocytes, highlighting the potential risk associated with excessive vitamin C intake. In the current study, the H_2_O_2_ levels, right before weaning in the vit-C group, were numerically higher than those in the control group. Elevated H_2_O_2_ levels could potentially increase the production of hydroxyl radicals, which are highly reactive molecules known for their capacity to cause extensive oxidative damage to oocytes [63]. Therefore, our findings emphasize that the dosage and potential side effects should be carefully considered when supplementing diets with vitamin C for reproductive management, especially in sows with large litters.

## 5. Conclusions

The utilization of a CCM as nesting material for sows with large litters was shown to have positive impact on NB, even on slatted flooring, which in turn, may have the potential to regulate the physiological and oxidative stress levels in these sows during lactation. This finding is indicative of the pivotal role of NB in alleviating stress, which influences oxidative stress management in sows with large litters. However, despite these positive effects, NB stimulation did not influence preovulatory follicular development, the reasons for which may be multifaceted. HVC supplementation in sows with large litters had a contrasting impact. It was negatively associated with the farrowing process, physiological stress, and follicle development. This adverse effect may be attributed to the potential risks associated with excessive vitamin C intake, which presumably leads to pro-oxidant effects and gastrointestinal disturbances. Therefore, managing the oxidative stress of highly prolific sows may necessitate the use of feed additives that can effectively counter oxidative stress without introducing adverse effects. Furthermore, understanding the intricate relationship between oxidative stress status and follicle development in sows with large litters requires further investigation.

## Figures and Tables

**Table 1:** Effects of CCM provision and HVC supplementation on salivary oxidative stress parameters of sows.

	Control	Vit-C	Mat	Mat + Vit-C	SEM	*p* Value		
*N*	9	8	10	8		Treatment	Mat ^1^	Vit-C ^2^
AOPP level (μmol/L) ^3^								
Day −4	9.38 ^a^	4.79 ^b^	5.41 ^b^	7.85 ^ab^	0.67	0.047	0.694	0.651
Day 1	7.54	7.33	6.11	5.43	0.67	0.521	0.175	0.709
Day 7	8.24 ^a^	3.10 ^ab^	2.81 ^b^	3.60 ^ab^	0.91	0.046	0.086	0.495
Day 28	2.62	2.03	4.14	7.14	0.69	0.292	0.093	0.905
Day −4–28	7.37 ^a^	4.83 ^ab^	4.66 ^b^	5.81 ^ab^	0.39	0.042	0.193	0.401
TEAC level (μM) ^3^								
Day −4	255.54	246.57	262.47	248.21	6.97	0.827	0.648	0.414
Day 1	210.33 ^ab^	242.50 ^ab^	180.46 ^b^	254.88 ^a^	10.07	0.040	0.361	0.014
Day 7	219.44	208.34	211.20	218.26	7.54	0.956	0.977	0.870
Day 28	187.62	226.34	204.55	178.89	11.73	0.464	0.405	0.971
Day −4–28	218.23	230.93	214.67	225.06	4.89	0.478	0.343	0.217
H_2_O_2_ level (μM) ^3^								
Day −4	5.51	9.67	24.36	8.11	3.69	0.285	0.142	0.787
Day 1	14.58	16.92	10.29	19.40	1.77	0.788	0.405	0.557
Day 7	9.04	11.01	8.36	5.91	1.25	0.786	0.828	0.408
Day 28	17.55	24.05	14.56	13.96	1.99	0.273	0.074	0.958
Day −4–28	12.23	15.76	13.58	12.09	1.09	0.614	0.896	0.985
TNF-α level (pg/mL) ^3^								
Day −4	63.28	99.21	61.11	63.40	9.84	0.570	0.414	0.244
Day 1	104.85	61.40	101.40	97.11	11.71	0.370	0.312	0.256
Day 7	51.74	47.17	60.35	43.03	4.52	0.662	0.858	0.268
Day 28	43.36	41.77	44.33	62.84	5.28	0.801	0.433	0.724
Day −4–28	65.81	61.20	66.80	66.60	4.41	0.834	0.630	0.652

^a,b^ Significantly different variables (*p* < 0.05). ^1,2^ Post-hoc contrast analysis depending on CCM provision or HVC supplementation. ^3^ All salivary samples were collected at 10:30, 4 days before parturition (late gestation) and 1, 7, and 28 days after parturition (lactation). AOPP, advanced oxidation protein products; CCM, coconut coir mat; H_2_O_2_: hydrogen peroxide; HVC, high-dose vitamin C; SEM, standard error of the mean; TEAC, Trolox equivalent antioxidant capacity; TNF-α, tumor necrosis factor-alpha.

**Table 2:** Effects of CCM provision and HVC supplementation on colostral hormones and Ig in sows.

	Control	Vit-C	Mat	Mat + Vit-C	SEM	*p* Value		
*N*	9	8	10	8		Treatment	Mat ^1^	Vit-C ^2^
Concentrations (pg/mL) ^3^								
Oxytocin	135.20	138.03	153.51	114.94	12.53	0.677	0.876	0.569
Prolactin	66.68	64.48	76.11	63.51	3.95	0.600	0.575	0.304
Concentrations (pg/mL) ^4^								
IgG	49.06	48.79	66.65	47.70	5.22	0.613	0.385	0.608
IgM	136.39	112.40	161.57	128.03	7.24	0.212	0.279	0.077
IgA	329.99	373.08	297.46	388.89	20.02	0.487	0.916	0.219

^1,2^ Post-hoc contrast analysis depending on CCM provision or HVC supplementation. ^3,4^ All colostrum samples were collected within 3 h after initiation of parturition. CCM, coconut coir mat; HVC, high-dose vitamin C; SEM, standard error of the mean; Ig, immunoglobulin.

**Table 3:** Effects of CCM provision and HVC supplementation on farrowing process and litter characteristics.

	Control	Vit-C	Mat	Mat + Vit-C	SEM	*p* Value		
*N*	9	8	10	8		Treatment	Mat ^1^	Vit-C ^2^
Farrowing process (min)								
Farrowing duration	205.64 ^c^	242.57 ^a^	203.56 ^c^	214.37 ^b^	16.64	<0.0001	0.008	<0.0001
Birth interval	14.40	17.05	13.97	15.47	1.19	0.277	0.474	0.078
Birth-to-udder touch interval	25.52 ^a^	25.55 ^a^	17.92 ^b^	21.97 ^ab^	1.46	0.003	0.002	0.045
Litter characteristics								
Total births, n	15.00	15.50	16.40	14.63	0.27	0.788	0.789	0.622
Live births, n	14.56	15.16	15.10	13.00	0.31	0.640	0.603	0.542
Stillbirths, n	0.44	0.38	1.30	1.63	0.20	0.053	0.006	0.965
Piglet birth weight, kg	1.50	1.44	1.39	1.53	0.03	0.270	0.773	0.358

^a,b,c^ Significantly different variables within the row (*p* < 0.05). ^1,2^ Post-hoc contrast analysis depending on CCM provision or HVC supplementation.

## Data Availability

Data are contained within the article.

## Abbreviations

AOPP: Advanced oxidation protein products; CCM: Coconut coir mat; H_2_O_2_: Hydrogen peroxide; HVC: High-dose vitamin C; Ig: Immunoglobulin; NB: Nest-building behavior; PG: Prostaglandin; ROS: Reactive oxygen species; TEAC: Trolox equivalent antioxidant capacity; TNF-α: Tumor necrosis factor-alpha.
